# Space Use, Physical Activity, Sleep Quality and Self-Rated Health Among Late-Life Asian Immigrants

**DOI:** 10.1093/geroni/igaa057.2969

**Published:** 2020-12-16

**Authors:** Wenjun Li, Elizabeth Proctor-Gray, Linda Churchill, Jie Cheng, Kevin Kane, Rachel Siden, Scott Crouter

**Affiliations:** 1 University of Massachusetts Medical School, Worcester, Massachusetts, United States; 2 The University of Tennessee, Knoxville, Tennessee, United States

## Abstract

Little is known about the health and health behaviors of non-English Speaking late-life Asian immigrants, which is attributable to language and cultural barriers to participate research. In our Healthy Aging and Neighborhood Study, we collected objective measures of space and time use, location- and time-specific physical activities using accelerometer and Global Positioning System devices, and self-reported sleep quality and health. We obtained 3,915 person-days of accelerometer readings from 511 participants including 43 Asians. Compared to non-Hispanic Whites, Asians had worse self-rated health and poorer vision, more medical conditions and physical limitations as measured by ADL and IADL. Asians had higher daily step counts overall and at home; high proportions of steps on sidewalk or street and senior centers, lower in fee-based outdoor recreational areas, less in restaurant and vehicle. Sleep quality did not differ significantly between Asians and Whites. Risk profiles differed between Asians and Whites, which warrants further investigation.

